# Intranasal delivery of cerium oxide nanozyme and superoxide dismutase in exosomes for Parkinson's disease therapy

**DOI:** 10.1016/j.mtbio.2026.103629

**Published:** 2026-09-02

**Authors:** Xinxin Shao, Kefei Li, Yanbo Wang, Xinai He, Boyu Xiong, Shengcai Yang, Quanshun Li

**Affiliations:** Key Laboratory for Molecular Enzymology and Engineering of Ministry of Education, School of Life Sciences, Jilin University, Changchun, 130012, China

**Keywords:** Exosome, Nanozyme, Superoxide dismutase, Parkinson's disease, Intranasal administration

## Abstract

The excessive reactive oxygen species (ROS) play an important role in the occurrence and progression of Parkinson's disease (PD). To investigate whether the antioxidant enzymes can scavenge the ROS level *in vivo* and treat PD efficiently, superoxide dismutase (SOD) was encapsulated into HEK293-derived exosomes to prepare SOD-loaded exosomes (SOD@EXO), and catalase (CAT)-like cerium oxide nanozyme (CeO_2_) was mixed with SOD@EXO to construct the formulation, namely SOD@EXO + CeO_2_. The formulation with cup-shaped morphology showed high SOD and CAT activities, which could scavenge the ROS level in a cascade manner. *In vitro* neuroprotective trials against SH-SY5Y cells revealed that SOD@EXO + CeO_2_ could efficiently prevent the neurotoxicity in 1-methyl-4-phenylpyridine-induced PD cell model. Moreover, the exosomes could be significantly accumulated in brain after the intranasal administration in comparison to the intravenous injection. Finally, the system was found to ameliorate the behavior disorder and relieve the inflammatory responses in 1-methyl-4-phenyl-1,2,3,6-tetrahydropyridine-induced PD mice model after the intranasal administration of SOD@EXO + CeO_2_. In a word, SOD@EXO + CeO_2_ could act in a cascade manner to scavege ROS, relieve the inflammatory response and improve the behavior disorder. Our results provide a new paradigm to construct the prevention and treatment strategy of dyskinesia diseases in central nervous system in future.

## Highlights

• SOD@EXO + CeO_2_ was prepared through mixing CeO_2_ nanozyme and SOD@EXO.
• SOD@EXO + CeO_2_ prevented the neurotoxicity in MPP ^+^ -induced PD cell model.
• Intranasal administration improved the accumulation of cargos in brain.
• SOD@EXO + CeO_2_ ameliorated behavior disorder and relieved inflammatory responses.

## Graphical abstract

**Figure:**
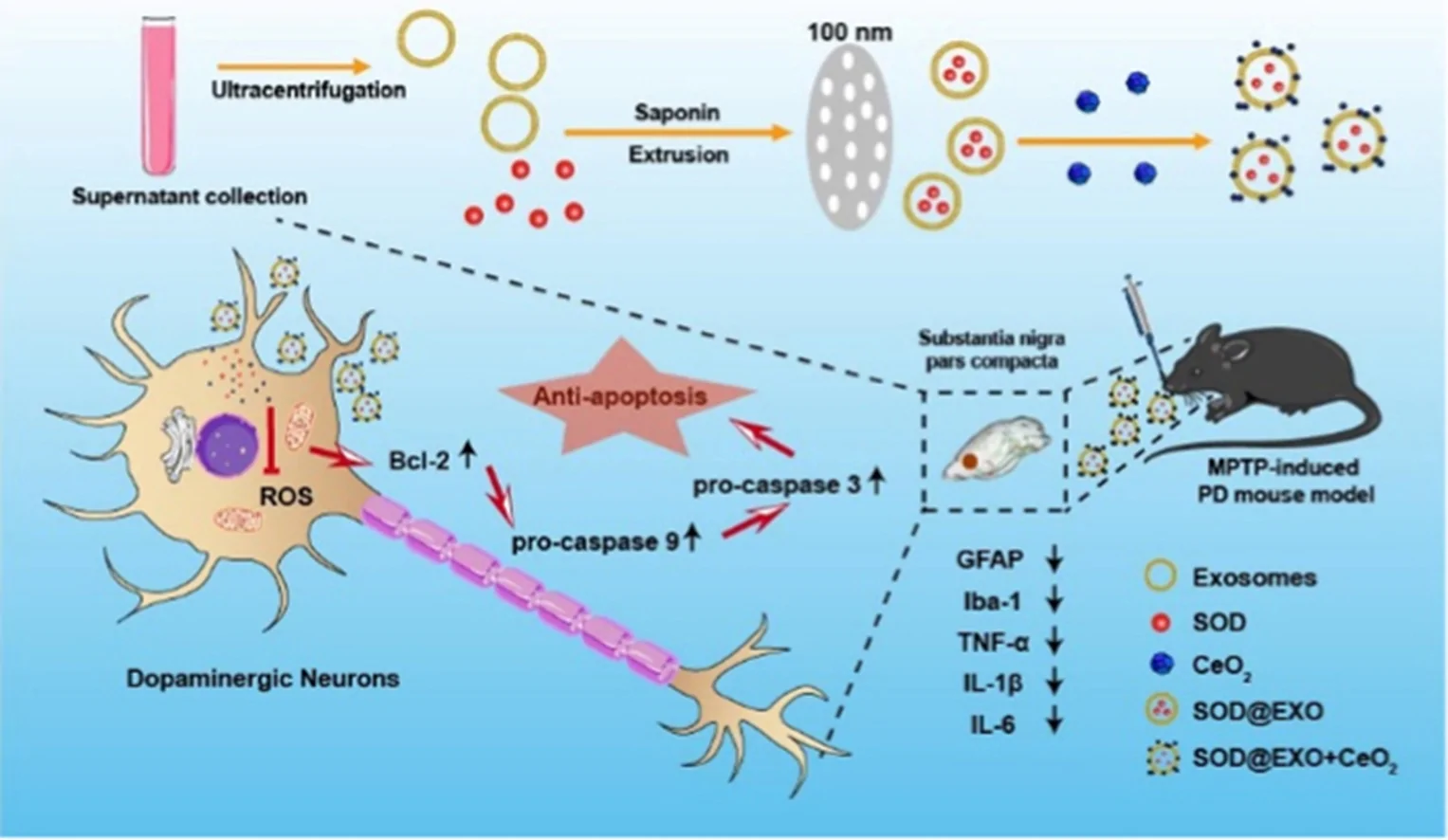

## 1 Introduction

Parkinson's disease (PD) is the most prevalent form of dyskinesia affecting the central nervous system (CNS) [1]. The principal pathological feature is the decreased activity of tyrosine hydroxylase and dopamine (DA) decarboxylase [2], which leads to the decreased number of dopaminergic neurons and concomitant depletion of DA in the striatum [3,4]. The occurrence and development of PD are highly dependent on the excessive level of reactive oxygen species (ROS) [5,6]. Thus, reducing the intracellular ROS level with antioxidant enzymes could alleviate the neuronal degeneration and damage, thereby providing a powerful tool for the PD treatment [7,8].

Superoxide dismutase (SOD) is an essential member of antioxidant enzymes and can scavenge superoxide anion (•O_2_^−^) *via* disproportionation reaction. In the organism's defense against oxidative stress of PD, SOD plays a key role in eliminating excessive ROS in neurons and reducing the peroxidation damage of brain tissue [9,10]. Except for SOD molecules, many nanozymes have been successfully developed with favorable anti-oxidant activity, high stability and low cost [[11], [12], [13]]. The representative nanozyme, cerium oxide nanoparticles (CeO_2_) can catalytically interact with hydrogen peroxide (H_2_O_2_) through redox cycles between Ce^3+^ and Ce^4+^ oxidation states, precisely imitating the actions of catalase (CAT) [[14], [15], [16], [17]]. In addition, CeO_2_ nanozymes offer excellent thermal and chemical stability, enabling cost-effective scale-up, long-term storage, and reproducible batch-to-batch performance, which are highly desirable for clinical translation in future. Previous research has demonstrated the CeO_2_ nanozyme's redox potential on the neuroprotection in PD treatment [10]. Further, the efficacy of CeO_2_ nanozyme as a neuroprotectant was identified to be associated with its ability to mimic CAT activity and scavenge ROS. Thus, SOD and CeO_2_ nanozyme could scavenge the ROS level in a cascade manner, where SOD first converts •O_2_^−^ to H_2_O_2_ and O_2_, and then the CAT-like CeO_2_ nanozyme catalyzes the conversion of produced H_2_O_2_ into H_2_O and O_2_. Thus, the ROS stress-induced damages could be mitigated by this action, which was essential to control the locomotor disorders and relieve the necroinflammation for PD therapy [18,19]. Liu et al. have prepared organic Se-doped carbon nitride quantum dots, which exhibited SOD/CAT-like enzymatic activity and effectively scavenged the ROS level to improve the motor dysfunction of MPTP-induced PD mice [20]. Based on these reports, we infer that the combination of SOD and CeO_2_ nanozyme may serve as an efficient strategy to achieve the PD treatment.

To improve the therapeutic efficacy of native enzyme, the delivery of enzyme has been successfully explored using nanoparticles, such as polymeric, inorganic, lipidic nanoparticles, and exosomes [21,22]. Among these nanoparticles, exosomes are good choices for the *in vivo* delivery of enzymes owing to their high biocompatibility and low immunogenicity [23,24]. In our previous work, SOD and chondroitinase ABC have been successfully loaded into exosomes, further indicating that the exosomes could be used as potential carriers for the delivery of therapeutic enzymes [25]. More importantly, exosomes have been demonstrated to penetrate the blood-brain barrier (BBB) and easily transport the cargos to CNS for the PD therapy [[26], [27], [28], [29]]. Except for the intrinsic characteristic of crossing BBB of exosomes, the intranasal administration of nanoparticles provides a direct anatomical pathway to CNS *via* the olfactory and trigeminal nerves, effectively bypassing the BBB. Moreover, the intranasal route could facilitate the delivery of cargos into the brain and avoid the systemic toxicity to normal tissues [30,31]. Batrakova et al. first reported the CAT-loaded exosomes, which exhibited excellent neuroprotective effects in PD mice model following the intranasal administration [32]. Meanwhile, the intranasal administration is a clinically established, patient-friendly method for drug delivery. It is non-invasive to circumvent the first-pass hepatic metabolism, and is easily self-administered to achieve the management of chronic conditions. Hence, the intranasal administration of exosomes encapsulating therapeutic agents will be a potential way to overcome BBB and treat the CNS disorders.

**Scheme 1:**
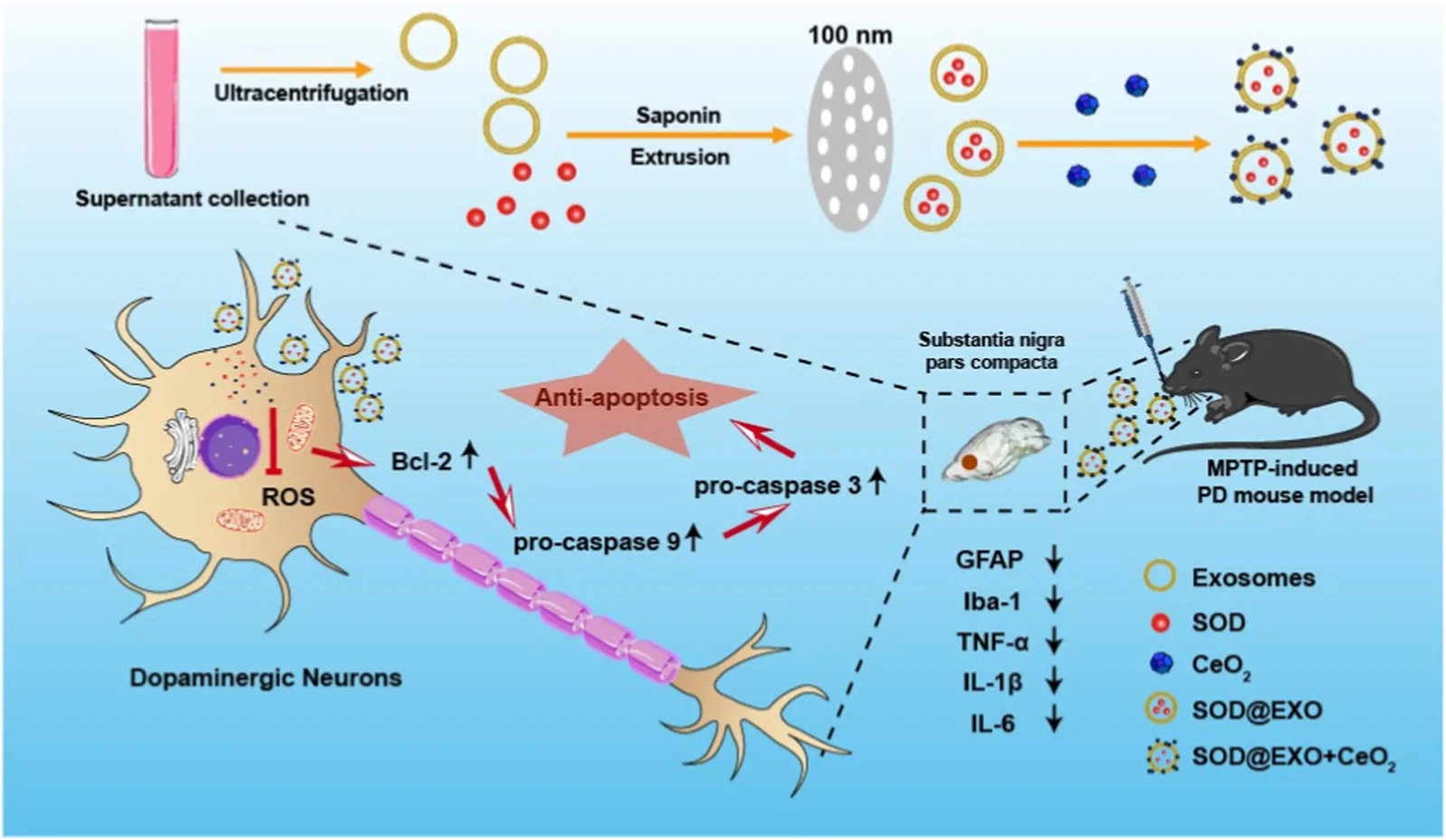
Construction of SOD@EXO + CeO_2_ and its therapeutic efficacy and mechanism analysis in MPTP-induced PD mice model.

Herein, an exosome-based delivery system of SOD (SOD@EXO) was constructed through the combination of mechanical extrusion and saponin permeabilization, which was mixed with CeO_2_ nanozyme to obtain SOD@EXO + CeO_2_ for PD therapy (Scheme 1). The obtained nanoparticles were characterized with antioxidant activity, cellular uptake and neuroprotective efficacy in the 1-methyl-4-phenylpyridine (MPP^+^)-induced PD cell model. *In vivo* biodistribution of exosomes was performed in C57BL/6 mice after the intravenous (*i.v.*) or intranasal (*i.n.*) administration. The therapeutic and anti-inflammatory efficacies of SOD@EXO + CeO_2_ were analyzed using 1-methyl-4-phenyl-1,2,3,6-tetrahydropyridine (MPTP)-induced PD mice model.

## 2 Materials and methods

### 2.1 Materials

SOD (Catalog No. BN20131) was purchased from BioDee Biotechnology (Beijing, China). Hematoxylin and eosin were obtained from Solarbio (Beijing, China). RPMI 1640 medium and Dulbecco's modified Eagle's medium (DMEM) were provided by Gibco (Grand Island, NY, USA) complemented with 10% fetal bovine serum (FBS) (Kangyuan Co., Beijing, China). PrimeScript™ RT-PCR kit with gDNA Eraser and SYBR® Premix Ex Taq™ PCR kit were acquired from TaKaRa (Dalian, China). 3-(4,5-Dimethylthiazol-2-yl)-2,5-diphenyltetrazolium bromide (MTT) was obtained from Amersco (Solon, OH, USA). Annexin V-FITC/PI apoptosis detection kit was purchased from Bestbio (Shanghai, China). LIVE/DEAD® Viability/Cytotoxicity kit was provided by Thermo Fisher Scientific (Waltham, MA, USA). The ROS assay kit and mitochondrial membrane potential assay kit with JC-1 were obtained from Beyotime Biotechnology (Nanjing, China). 1,1′-Dioctadecyl-3,3,3′,3′-tetramethylindotricarbocyanine iodide (DIR, Catalog No. D12731) and 1,1′-dioctadecyl-3,3,3′,3′-tetramethylindodicarbocyanine perchlorate (DID, Catalog No. D307) were acquired from Invitrogen Life Technologies (Carlsbad, CA, USA). MPP^+^ and MPTP were obtained from Sigma-Aldrich (St. Louis, MO, USA).

### 2.2 Preparation of SOD@EXO + CeO_2_

CeO_2_ nanozyme was prepared according to the previous reports [33,34]. Briefly, a mixture solution of cerium (III) acetate (1 mmol, 0.43 g), oleylamine (12 mmol, 3.25 g), and xylene (15 mL) was subjected to vigorous stirring at 25 °C for 12 h. The solution was then heated to 90 °C at a rate of 2 °C/min, and 1 mL of deionized water was rapidly injected to initiate a sol-gel reaction. The mixture was stirred at 90 °C for 3 h and subsequently cooled to 25 °C. Finally, the sample was washed with acetone and centrifuged at 8000 rpm for 8 min to obtain CeO_2_ nanozyme.

Next, SOD-loaded exosomes (SOD@EXO) were prepared according to our previous reports [25,35]. Then the obtained SOD@EXO was mixed with CeO_2_ nanozyme (25.0 μg/mL) to obtain SOD@EXO + CeO_2_. The morphology of SOD@EXO + CeO_2_ was detected by a high-resolution transmission electron microscope at 200 kV (HR-TEM, JEM 2100F, Tokyo, Japan). The particle size, polydispersity index (PDI) and zeta potential of SOD@EXO + CeO_2_ was detected by Nano ZS90 Zetasizer (Malvern Instruments, UK). The stability of SOD@EXO + CeO_2_ in PBS and 10% FBS was evaluated by measuring the particle size and PDI values using Nano ZS90 Zetasizer.

### 2.3 Enzymatic activity

The antioxidant activities of SOD, CeO_2_, SOD@EXO or SOD@EXO + CeO_2_ were evaluated using superoxide dismutase activity assay kit (SOD, S0101, Beyotime, China) and catalase activity assay kit (CAT, S0051, Beyotime, China). SOD activity was determined according to our previous reports [25,35]. CAT activity was measured based on that the unconverted H_2_O_2_ could react with the probe to produce a red product after the decomposition of H_2_O_2_ by the samples.

### 2.4 In vitro BBB permeability

To construct a compact monolayer, human umbilical vein endothelial cells HUVEC (5 × 10^5^ cells/well, 500 μL/well) were seeded into the upper Transwell chamber (0.33 cm^2^, 0.4 μm pore size, Corning Costar®, NY, USA), and the basolateral Transwell chamber was filled with 10% FBS-harboring medium (700 μL) and incubated at 37 °C [36]. The transendothelial electrical resistance (TEER) value of HUVEC cells was monitored by an epithelial volt-ohmmeter (EVOM, WPI) to monitor the tightness of the monolayer. The *in vitro* BBB cell model was considered to be successful when the TEER value was more than 200 Ω cm^2^. The TEER was calculated according to the following equation:

TEER (Ω·cm^2^) = (T_sample_-T_0_) × A

in which T_sample_ (Ω) and T_0_ (Ω) were the resistance value of sample and blank, respectively, and A (cm^2^) was the surface area of the cell monolayer.

To evaluate the transportation efficiency of native SOD and SOD@EXO across the BBB cell model, the upper chambers were incubated with FITC-SOD (100 μg/mL) or FITC-SOD@EXO (100 μg/mL on FITC-SOD basis) for 6 h, and the basolateral medium was collected to detect the fluorescent intensity by Shimadzu RF-5301 fluorescence spectrometer (Kyoto, Japan). The apparent permeability coefficient (P_app_) was calculated according to the following equation:

P_app_ (cm/s) = (Q/t) × (1/A) × (1/C)

in which Q (μg) was the total amount of FITC in the basolateral chamber, C (μg/cm^3^) was the initial concentration of FITC solution, t (s) was the incubation time, and A (cm^2^) was the surface area of cell monolayers.

### 2.5 In vivo biodistribution studies

Male C57BL/6 mice (4-6 weeks old) were purchased from Vital River Laboratory Animal Technology Co. Ltd. (Beijing, China). All the animal studies were approved by the Institutional Animals Ethics Committee of Jilin University (license No. SYXK(JI)2021-0141 and YNPZSY2026013).

Exosomes (2.0 mg) were incubated with DIR dye at 25 °C for 15 min, and the mixture was ultracentrifuged (100,000×*g*, 90 min) to remove free DIR. The precipitate was collected and resuspended in PBS to obtain the DIR-labeled exosomes (DIR-EXO). To trace the *in vivo* fluorescence of DIR-EXO, the mice were intravenously (*i.v.*) or intranasally (*i.n.*) administered with DIR-EXO (20.0 μg/mouse based on DIR) using the ones injected *via* tail vein with free DIR (20.0 μg/mouse) as a control. Finally, the mice were sacrificed after 1, 3 and 6 h, and major tissues (heart, liver, spleen, lung, kidney and brain) were excised and imaged by IVIS Spectrum *in vivo* imaging system (Caliper Life Sciences, Hopkinton, MA, USA).

### 2.6 Therapeutic efficacy of SOD@EXO + CeO_2_ in MPTP-induced PD mice model

The MPTP solution was used to induce the PD mice model as described previously [37]. In brief, male C57BL/6 mice (20-22 g) were randomly divided to seven groups (n = 5 per group): Group 1, untreated healthy mice (Normal); Group 2, MPTP; Group 3, MPTP + CeO_2_ (*i.v.*); Group 4, MPTP + SOD (*i.v.*); Group 5, MPTP + SOD@EXO (*i.v.*); Group 6, On DayThe dosage of SOD, SOD@EXO and SOD@EXO + CeO_2_ were 600 U/kg on SOD basis, while the dosage of CeO_2_ was 100 U/kg. Except for Group 1, all the mice were intraperitoneally injected with MPTP solution (35 mg/kg per day) for consecutive 5 days from Day 0 to Day 4. For the therapeutic efficacy studies, the mice were intravenously (*i.v.*) or intranasally (*i.n.*) administered with different formulations every other day for 6 times after finishing the injection of MPTP. Behavioral test was conducted every other day from Day 4 to Day 16, and then the mice were euthanized on Day 16 for further measurement. The climbing pole test was carried out to trace the locomotory behavior of mice, in which the mice were placed on the side of a rough-surfaced pole (100-cm length with 1-cm diameter). The time of mice climbing from one side of the pole to the other was measured, with 3 times for each mouse.

### 2.7 Measurement of oxidative stress indicators

Briefly, the mice were sacrificed on Day 16, and the substantia nigra pars compacta (SNpc) homogenates were obtained through the centrifugation at 10,000×*g* for 15 min (4 °C). The malondialdehyde (MDA) content and SOD activity in SNpc were determined according to the manufacturers' instructions (MDA, A003, Jiancheng Bioengineering Institute, Nanjing, China; SOD, S0101, Beyotime, Nanjing, China). To detect the ROS level, SNpc was placed on a 300-mesh nylon net and gently rubbed with a spatula, and the collected cell suspension was centrifuged (2000 rpm, 10 min) to obtain the cells. The ROS level in SNpc was determined according to the manufacturer's protocol (S0033, Beyotime, Nanjing, China).

### 2.8 Measurement of dopamine (DA)

The mice were sacrificed on Day 16, and the paired striatal were separated, homogenized and centrifuged (10,000×*g*, 15 min) at 4 °C to obtain striatum homogenates. The DA level was measured by enzyme-linked immunosorbent assay (ELISA) (Sangon Biotech, Shanghai, China).

### 2.9 Statistical analysis

The statistical analysis was conducted using the GraphPad Prism 5.0 (San Diego, CA, USA). A Student's t-test (unpaired, two-tailed) was employed in the comparison of different data between two groups. One-way ANOVA with Tukey's post hoc test and two-way ANOVA analysis with Bonferroni post hoc test were performed to determine the statistical significance of data between multiple groups. *P* value lower than 0.05 was the significance threshold (*P < 0.05, **P < 0.01, ***P < 0.001, ****P < 0.0001).

## 3 Results and discussion

### 3.1 Construction and characterization of SOD@EXO + CeO_2_

**Fig. 1:**
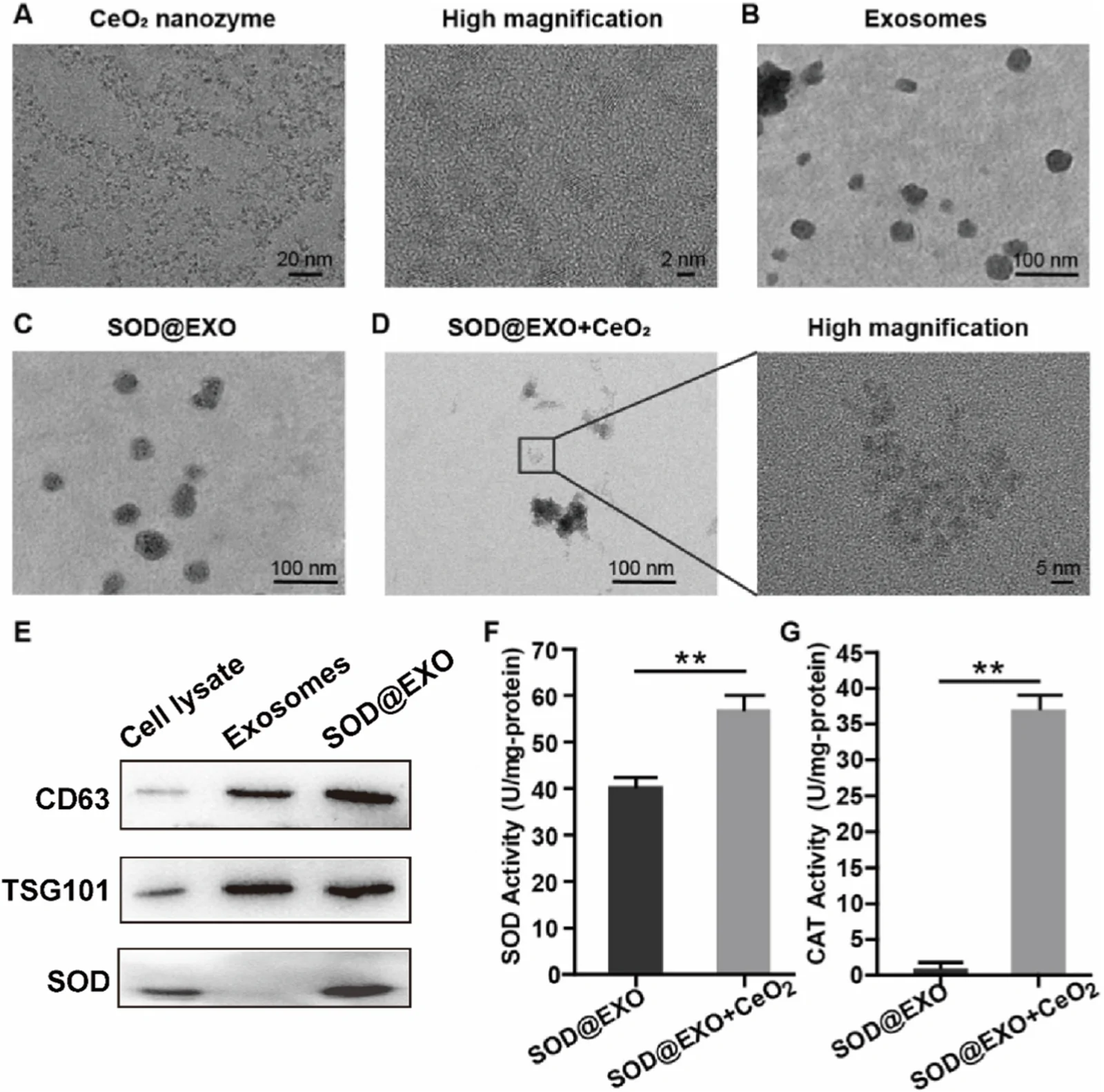
The HR-TEM images of (A) CeO_2_ nanozyme, (B) Exosomes, (C) SOD@EXO and (D) SOD@EXO + CeO_2_. (E) Western blotting analysis for the expression of CD63, TSG101 and SOD in cell lysate, exosomes and SOD@EXO. (F) SOD and (G) CAT activity of SOD@EXO and SOD@EXO + CeO_2_, in which data were presented as mean value ± SD of triplicate experiments (n = 3, **P < 0.01).

Firstly, CeO_2_ nanozyme was prepared according to the previous work [33]. CeO_2_ nanozyme has attracted great attention in the treatment of neurodegenerative diseases due to its potent ROS-scavenging and anti-inflammatory properties, as well as its superior stability and scalable production [38]. As shown in Fig. 1A, HR-TEM images of CeO_2_ nanozyme showed a uniformly spherical structure with an average diameter of 3 nm. CeO_2_ nanozyme exhibited an obvious lattice stripe-like structure, which was consistent with the previously reported result [33]. Cytotoxicity analysis of CeO_2_ nanozyme showed a dose-dependent manner using SH-SY5Y cells as a model (Fig. S1). Previous report demonstrated that the increasing use of CeO_2_ nanozyme presented potential health risk and safety concerns for the future [39]. It was worth noting that most of the published research examining the health effects of cerium on human health was focused on a microscale rather than the nanoscale CeO_2_ nanozyme [40]. As the cell viability values were measured to be over 90% at the concentrations of CeO_2_ nanozyme <25 μg/mL, this concentration was employed in the further experiments.

The antioxidant enzyme SOD-loaded exosomes (SOD@EXO) were prepared according to our previous work [25,35]. The exosomes secreted from HEK293 cells possessed an average diameter of 100.6 ± 0.9 nm, which was similar with SOD@EXO (96.8 ± 2.5 nm) (Fig. S2A and Table S1). In addition, both exosomes and SOD@EXO showed typically cup-shaped morphology (Fig. 1B and C), which was consistent with our previous reports [25,35]. Then, SOD@EXO was physically mixed with CeO_2_ nanozymes to prepare the formulation SOD@EXO + CeO_2_, and its HR-TEM image displayed that CeO_2_ nanozymes were adsorbed on the surface of exosomes or freely dispersed in the formulation, which did not change the membrane composition of exosomes (Fig. 1D). XRD analysis was performed for CeO_2_ nanozyme, SOD@EXO and SOD@EXO + CeO_2_ in PBS. As shown in Fig. S3, SOD@EXO exhibited no characteristic diffraction peaks within the 2θ range of 10°-80°. However, SOD@EXO + CeO_2_ exhibited a distinct diffraction peak assignable to the (111) crystal plane of CeO_2_, confirming the presence of CeO_2_ nanozyme in its crystalline form in the formulation. The hydrodynamic diameter and zeta potential of SOD@EXO + CeO_2_ were measured to be 103.2 ± 1.7 nm and −19.6 ± 3.7 mV, respectively (Fig. S2 and Table S1). As shown in Fig. S4, the average particle size remained stable at approximately 105.7 nm, and the PDI values were consistently below 0.3 throughout the incubation of 120 h, indicating that SOD@EXO + CeO_2_ did not undergo significant aggregation or degradation in PBS and 10% FBS. These results demonstrated that the SOD@EXO + CeO_2_ nanoparticles possessed favorable serum stability, supporting its potential use for intravenous administration.

It was widely accepted that CD63 and TSG101 were prominent protein markers on the surface of exosomes [41]. Western blotting results showed high expression of CD63 and TSG101 in SOD@EXO and EXO, indicating that the SOD loading did not change the intact structure of exosomes (Fig. 1E). Furthermore, a distinct SOD band was present in SOD@EXO but barely detectable in EXO, which further verified the high purity and structural integrity of the isolated EXO and successful enzyme loading. In contrast, the expression of these markers was very low in the cell lysate. Different concentrations of SOD were then used as reference standards in Western blotting analysis (Fig. S5), and the amount of SOD in SOD@EXO was 416 ng, with a calculated drug loading content (DLC, %) of 12.18%. The SOD and CAT enzymatic activities of SOD@EXO and SOD@EXO + CeO_2_ were evaluated through the corresponding detection kits. As shown in Fig. S6, the natural enzyme SOD had a robustly specific SOD activity. CeO_2_ nanozyme displayed predominant CAT-mimetic activity of 828 U per mg CeO_2_, accompanied by mild SOD-like activity, which were in accordance with the previous reports [33,42]. The formulation SOD@EXO + CeO_2_ exhibited much higher SOD and CAT activities than those of SOD@EXO, especially for the improved SOD activity after mixing with CeO_2_ nanozyme (Fig. 1F and G). These results demonstrated that the system SOD@EXO + CeO_2_ exhibited ideal antioxidant effects through the combination of SOD and CAT activities.

### 3.2 BBB penetration and cellular uptake of SOD@EXO + CeO_2_

**Fig. 2:**
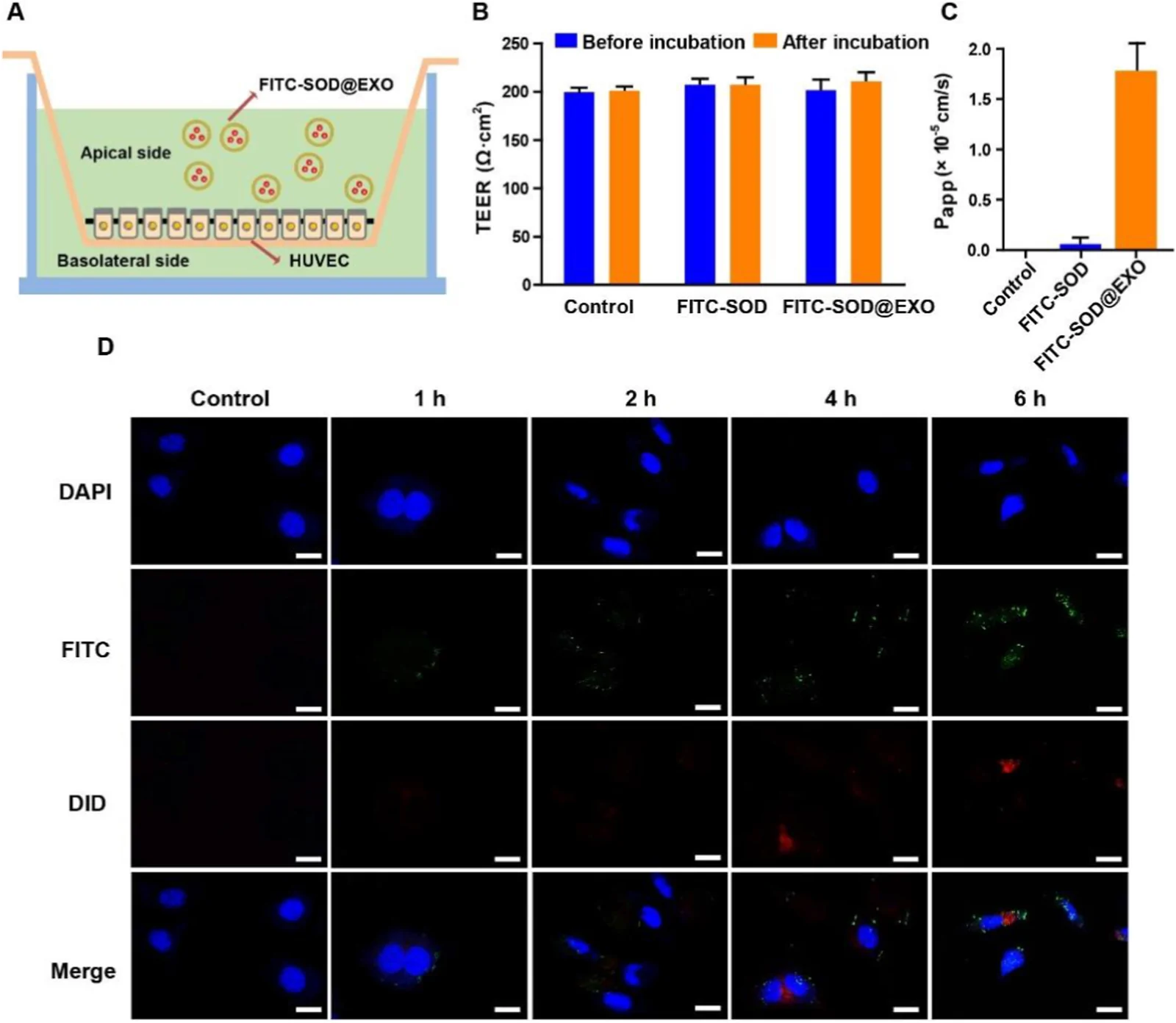
(A) The schematic illustration of the *in vitro* cellular internalization and BBB permeability of FITC-SOD@EXO. (B) TEER values before and after the incubation with FITC-SOD and FITC-SOD@EXO for 6 h (n = 3). (C) P_app_ values of FITC-SOD and FITC-SOD@EXO measured by a fluorescence spectrometer (n = 3). (D) CLSM images of SH-SY5Y cells after the incubation with FITC-SOD@DID-EXO for 1, 2, 4 and 6 h. The SOD and exosomes were labeled with FITC (green) and DID (red), respectively, and the nuclei were stained with DAPI (blue). Scale bar: 10 μm. (For interpretation of the references to colour in this figure legend, the reader is referred to the Web version of this article.)

BBB is not only an effective barrier to protect CNS, but also a barrier to hamper the transport of therapeutic drugs into brain [36]. The BBB penetration ability of FITC-labeled SOD (FITC-SOD) and FITC-SOD@EXO was evaluated. As shown in Fig. 2A, the formation of HUVEC monolayer cell onto the upper Transwell membrane was used to construct the *in vitro* BBB cell model. As shown in Fig. 2B, TEER value of HUVEC cells was up to 200 Ω cm^2^, indicating the tight junctions of this BBB model were integrous. After the incubation with FITC-SOD or FITC-SOD@EXO for 6 h, the TEER values did not change significantly, indicating no compromise in the BBB integrity (Fig. 2B). Moreover, the fluorescent intensity of medium in the basal chamber was measured to assess the permeability of FITC-SOD@EXO. The P_app_ value of FITC-SOD@EXO was 30-fold higher than that of FITC-SOD (1.78 ± 0.45 × 10^−5^ cm/s *vs* 0.06 ± 0.07 × 10^−5^ cm/s), indicating that SOD@EXO traversed BBB more efficiently (Fig. 2C). These findings were consistent with the published data for the BBB permeability of exosomal vehicles [32,43]. To study the cellular uptake of SOD@EXO against SH-SY5Y cells, exosomes and SOD were labeled with lipophilic membrane dye DID (Red) and FITC (Green), respectively, and the intracellular distribution of FITC-SOD@DID-EXO was visualized after the incubation for 1, 2, 4 or 6 h by confocal laser scanning microscopy (CLSM). The intensity of green or red fluorescence increased with the incubation time, and the highest cellular uptake of FITC-SOD@DID-EXO could be achieved after the incubation for 6 h (Fig. 2D). The high cellular internalization provided us the reason for the favorable penetration ability of SOD@EXO in the *in vitro* BBB cell model.

### 3.3 In vitro neuroprotective efficacy and mechanism of SOD@EXO + CeO_2_

**Fig. 3:**
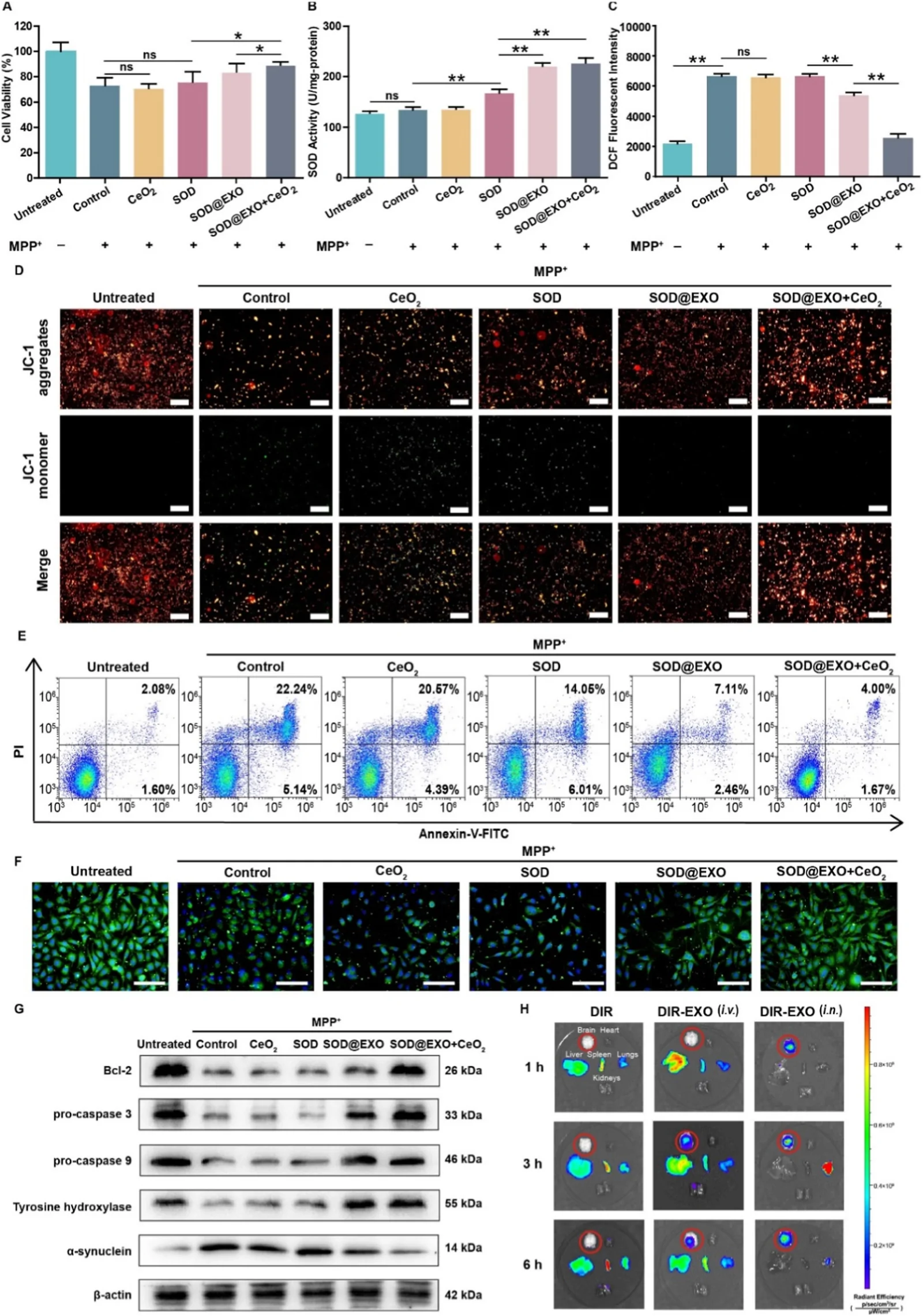
(A) The cell viability (n = 6) and (B) intracellular SOD activity (n = 3) of SH-SY5Y cells pretreated with different groups for 6 h in the MPP^+^-induced PD cell model (*P < 0.05, **P < 0.01, ns, not significant). (C) The cytofluorimetric quantitation of DCF to determine the ROS level in the MPP^+^-induced PD cell model (n = 3, **P < 0.01, ns, not significant). (D) The mitochondrial membrane potential of SH-SY5Y cells pretreated with different groups in the MPP^+^-induced PD cell model. Scale bar: 200 μm. (E) Representative scatter diagram of cell apoptosis based on Annexin V-FITC/PI staining in flow cytometry assay. (F) Immunofluorescence staining of tyrosine hydroxylase (green) in the MPP^+^-induced PD cell model, and the nuclei were stained with DAPI (blue). Scale bar: 200 μm. (G) Western blotting analysis for the expression of apoptosis-related proteins (Bcl-2, pro-caspase 3 and pro-caspase 9) and PD-related proteins (tyrosine hydroxylase and α-synuclein) after the treatment with different formulations in the MPP^+^-induced PD cell model. (H) *Ex vivo* fluorescent images of brain, heart, liver, spleen, lungs and kidneys at 1, 3 and 6 h after the intravenous (*i.v.*) or intranasal (*i.n.*) administration of DIR-EXO. The brain tissues were circled in red lines. (For interpretation of the references to colour in this figure legend, the reader is referred to the Web version of this article.)

Here, MPP^+^ (2 mM) was used to treat SH-SY5Y cells to construct the *in vitro* PD cell model [37], after which MTT and live/dead staining assays were conducted to evaluate the neuroprotective efficacy of SOD@EXO + CeO_2_. For MTT assay, the cell viability was only 73% under the MPP^+^ treatment without any protection, indicating the successful construction of PD cell model (Fig. 3A). Both free CeO_2_ nanozyme and native SOD could not prevent the MPP ^+^ -induced damage. The treatment with SOD@EXO + CeO_2_ achieved 89% of cell viability exhibiting the most effective neuroprotection, which was mainly caused by the combined antioxidant effect of SOD and CeO_2_ nanozyme. For live/dead staining assay, the MPP^+^-treated cells showed stronger red fluorescence than that of untreated healthy group (Fig. S7), in which live and dead cells were stained green and red, respectively. In contrast, the SOD@EXO + CeO_2_ pretreatment group showed fewer dead cells compared to other groups. All these data suggested that SOD@EXO + CeO_2_ could be used to act as a shield against the MPP ^+^ -induced neurotoxicity.

The intracellular SOD activity and ROS level of SH-SY5Y cells were evaluated in MPP^+^-induced PD cell model after the treatment with different formulations. As shown in Fig. 3B, the MPP^+^ induction did not exert an influence on the intracellular SOD activity in comparison to the untreated healthy group. In contrast, native SOD could enhance the intracellular enzymatic activity. Notably, the SOD activities of SH-SY5Y cells after the pretreatment with SOD@EXO and SOD@EXO + CeO_2_ were 1.32-fold and 1.35-fold higher than that of native SOD, respectively. The ability of SOD@EXO + CeO_2_ to scavenge the intracellular ROS level was further investigated in SH-SY5Y cells by DCFH-DA probe using flow cytometry. Compared with the untreated healthy cells, MPP^+^ prominently elevated the intracellular ROS level (Fig. S8 and 3C). The cells pretreated with CeO_2_ nanozyme or native SOD showed similar DCF fluorescent intensity after the MPP ^+^ stimulation, indicating that these two components was unable to scavenge the excessive ROS level. In contrast, the ROS level in SH-SY5Y cells with SOD@EXO pretreatment was much lower than that of native SOD group, which was probably caused by that the SOD loading in exosomes was beneficial to achieve its cellular uptake and execute its anti-oxidative effect. Meanwhile, the exosomes were useful to improve the cytosolic stability of SOD molecules, thereby obtaining ideal catalytic activity in the cytosol. More importantly, the concentration of SOD@EXO (6 U/mL on SOD basis) was used according to the previous report [25], which could effectively scavenge the intracellular ROS of lipopolysaccharide-activated BV2 cells. Following the preincubation with SOD@EXO + CeO_2_ for 6 h, the ROS level was most effectively inhibited in the MPP^+^-induced PD cell model, indicating that the combination of SOD and CeO_2_ nanozyme could achieve the highest ability of ROS scavenging. All these findings confirmed that SOD@EXO + CeO_2_ could significantly enhance the intracellular SOD and CAT activities and eliminate the intracellular ROS level.

To investigate the protective mechanism of SOD@EXO + CeO_2_ against the MPP ^+^ -induced neurotoxicity in the PD cell model, mitochondrial membrane potential assay was conducted, in which JC-1 (an indicator of mitochondrial membrane potential) was utilized to assess the mitochondrial function [44]. JC-1 dye aggregated and emitted red fluorescence in high mitochondrial membrane potential, while it existed as monomers and showed green fluorescence in lower potential. Clearly, the MPP^+^ treatment group showed a robust manifestation of green fluorescence, thereby signifying the status of lower mitochondrial membrane potential (Fig. 3D). Compared with the native SOD group, weaker green fluorescence was detected in the SOD@EXO pretreatment cells, proving that the delivery system SOD@EXO could prevent the mitochondrial dysfunction. The phenomenon was mainly attributed to the higher ROS scavenging ability owing to the exosomes-mediated internalization of SOD molecules. The red fluorescence in the SOD@EXO + CeO_2_ treatment group was further enhanced, reaching a level close to the untreated healthy group. As the intracellular ROS level could lead to the decreased mitochondrial membrane potential, the lower ROS level of SOD@EXO + CeO_2_ group accounted for the strong red fluorescent intensity. These results suggested that SOD@EXO + CeO_2_ could inhibit the MPP ^+^ -induced mitochondrial dysfunction of SH-SY5Y cells, and the decreased mitochondrial membrane potential of depolarized mitochondria was considered as the beginning of apoptosis.

Further, SH-SY5Y cells were stained with Annexin V-FITC/PI to investigate the apoptotic effect of SOD@EXO + CeO_2_ in MPP ^+^ -induced PD cell model (Fig. 3E). The apoptosis ratio of MPP^+^ treatment group was significantly higher than that of untreated cells (27.38% *vs* 3.68%). The CeO_2_ nanozyme exhibited a similar apoptotic ratio, demonstrating no apoptotic protection ability, in accordance with the low mitochondrial membrane potential after its treatment. The ratio dramatically decreased through the pretreatment with SOD@EXO + CeO_2_ (5.67%), implying its superior alleviation efficacy for the MPP^+^-induced neurotoxicity. Flow cytometric analysis consistently revealed that the percentage of apoptotic cells in SOD@EXO + CeO_2_ group was significantly lower than SOD and SOD@EXO groups. To ascertain the neuroprotective mechanism of SOD@EXO + CeO_2_, the expression levels of mitochondrial apoptosis pathway-related proteins (Bcl-2, pro-caspase 3 and pro-caspase 9) were assayed by Western blotting (Fig. 3G). Clearly, the levels of these proteins have been significantly reduced when the cells were treated with MPP^+^ in comparison to the untreated group. Meanwhile, the expression levels of Bcl-2, pro-caspase 3 and pro-caspase 9 increased remarkably after the preincubation with SOD@EXO and SOD@EXO + CeO_2_ for 6 h, much higher than CeO_2_ and SOD groups. All these findings revealed that SOD@EXO + CeO_2_ could prevent the MPP ^+^ -induced neurotoxicity in PD cell model by inhibiting the cell apoptosis.

Tyrosine hydroxylase is mainly found in dopaminergic neurons which reflects the growth status of nerve cells, and α-synuclein secreted by dopaminergic neurons is another key pathologic hallmark of PD [45]. Therefore, the expressions of tyrosine hydroxylase and α-synuclein was evaluated by Western blotting to study the neuroprotective ability of different formulations (Fig. 3G). After the MPP^+^ stimulation, the expression of α-synuclein was significantly upregulated, while the level of tyrosine hydroxylase was inhibited, indicating the poor growth status of SH-SY5Y cells and the successful construction of *in vitro* PD cell model. The expressions of α-synuclein and tyrosine hydroxylase between CeO_2_ or SOD treatment groups and the MPP ^+^ -induced group were of no significant differences. However, higher α-synuclein expression and lower tyrosine hydroxylase level could be detected after the treatment with SOD@EXO. Among all the groups, SOD@EXO + CeO_2_ showed the highest expression of tyrosine hydroxylase and the lowest level of α-synuclein, which recovered to the levels close to the untreated healthy group. Moreover, immunofluorescence analysis was used to measure the expression level of tyrosine hydroxylase in which green fluorescence represented its level (Fig. 3F). Similarly, the green fluorescent intensity was significantly upregulated after the preincubation with SOD@EXO + CeO_2_, and the results were consistent with Western blotting analysis of tyrosine hydroxylase.

### 3.4 In vivo biodistribution of DIR-EXO

To identify the *in vivo* biodistribution of DIR-EXO after *i.v.* or *i.n.* administration, C57BL/6 mice were subjected for *ex vivo* optical imaging. Here these mice were assigned into three groups: free DIR (*i.v.*), DIR-EXO (*i.v.*) and DIR-EXO (*i.n.*). The mice were euthanized at predetermined time intervals (1, 3 and 6 h), and major organs (heart, liver, spleen, lungs, kidneys and brain) were excised and imaged by an IVIS Spectrum *in vivo* imaging system (Fig. 3H and S9). Free DIR did not accumulate in the brain at all the time points, proving that the dye could not cross BBB. For the DIR-EXO (*i.v.*) group, no fluorescence could be detected in the brain at 1 h post-injection, and the fluorescent intensity significantly increased after 3 or 6 h. In contrast, a rapid accumulation of DIR in the brain could be observed after the intranasal administration of DIR-EXO. At 1 h post-injection, the mice treated with DIR-EXO (*i.n.*) showed stronger fluorescence in the brain compared to those treated with DIR-EXO (*i.v.*). Similar results could be obtained at 3 or 6 h post-injection of DIR-EXO (*i.n.*). Notably, DIR-EXO (*i.n.*) did not distribute in other organs such as liver and spleen. A phase I/II clinical trial (ClinicalTrials.gov NCT04388982) also demonstrated that the intranasal administration of exosomes at a dose of 4 × 10^8^ particles was safe and well tolerated in the Alzheimer's disease therapy [46]. More importantly, Batrakova et al. reported that the intranasal administration of empty exosomes did not influence the number of DA neuron and the microglial activation in comparison to the PBS-treated healthy mice, suggesting the absence of neurotoxicity of exosomes in the brain [32]. These results clearly showed the potential application of exosomes as a vehicle for the nose-to-brain delivery into CNS *via* olfactory bulb. In future, the wide application of exosomes as carriers requires to explore new methods to achieve scalability, standardization, and consistency of manufacturing. Meanwhile, a considerable amount and long-term storage of exosomes should also be addressed for the emergency situations.

### 3.5 In vivo therapeutic efficacy of SOD@EXO + CeO_2_

**Fig. 4:**
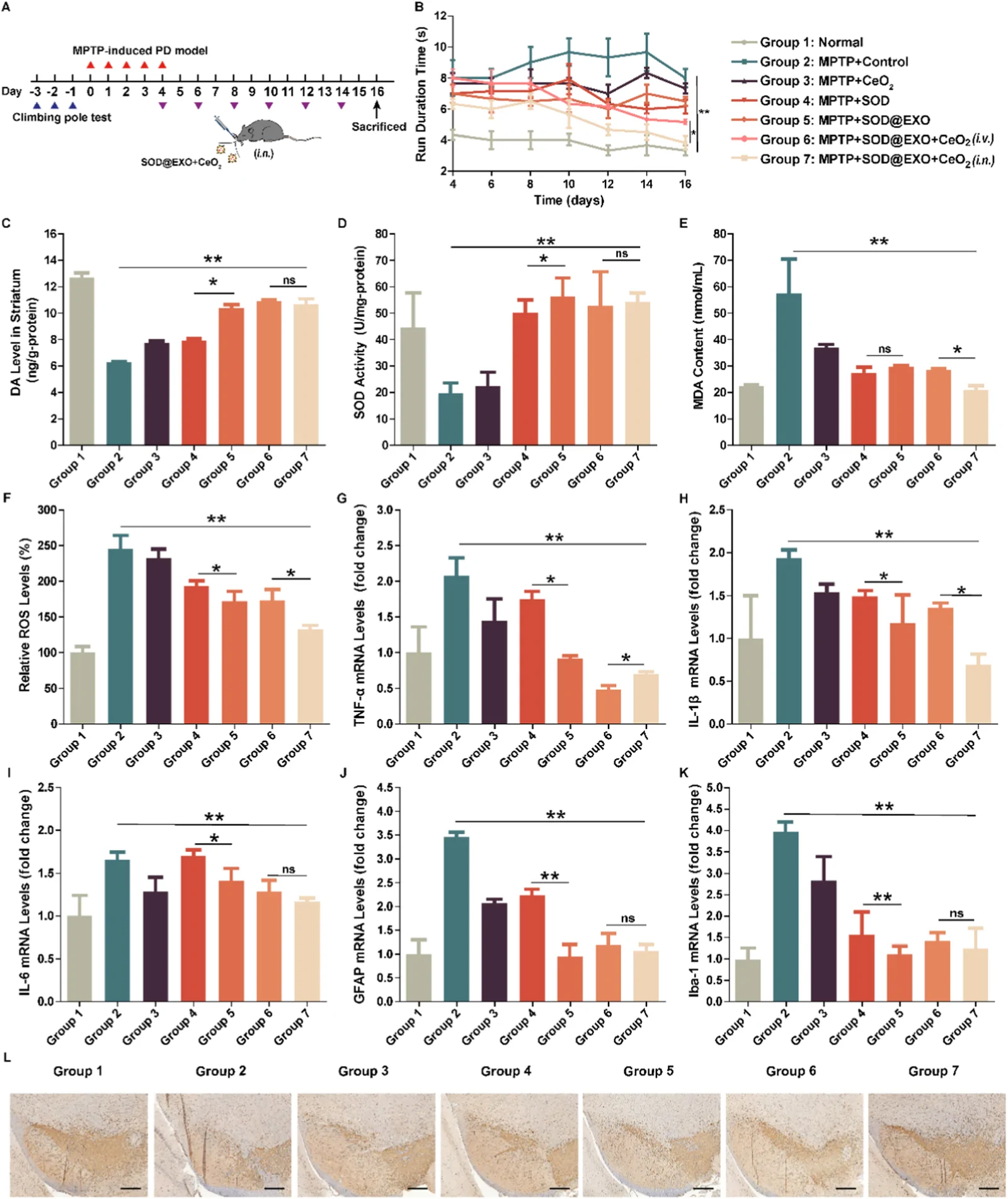
(A) Therapeutic schedule in the MPTP-induced PD mice model. (B) Run duration time of mice measured from Day 4 to evaluate the PD recovery (n = 5, *P < 0.05, **P < 0.01). (C) The DA level in striatum after different treatments on Day 16 (n = 5, *P < 0.05, **P < 0.01, ns, not significant). (D) SOD activity, (E) MDA content and (F) ROS level in SNpc after different treatments on Day 16 (n = 5, *P < 0.05, **P < 0.01, ns, not significant). The levels of (G) TNF-α, (H) IL-1β, (I) IL-6, (J) GFAP and (K) Iba-1 in SNpc after different treatments (TNF-α: tumor necrosis factor-α, IL-6: interleukin 6, IL-1β: interleukin 1β, Iba-1: ionized calcium binding adapter molecule 1, GFAP: glial fibrillary acidic protein; n = 5, *P < 0.05, **P < 0.01, ns, not significant). (L) Representative immunohistochemical staining images of tyrosine hydroxylase in SNpc. Scale bar: 300 μm.

The effective prevention ability in MPP^+^-induced PD cell model and high accumulation in the brains supported further investigations to evaluate the *in vivo* therapeutic efficacy of SOD@EXO + CeO_2_. Considering the different accumulation of DIR-EXO in the brain using two administration routes, the therapeutic efficacy of SOD@EXO + CeO_2_ in male C57BL/6 mice was assessed after the *i.v.* or *i.n.* administration at the same dose. These mice were randomly divided into 7 groups (n = 5) on Day 0: Group 1, untreated healthy mice (Normal); Group 2, MPTP; Group 3, MPTP + CeO_2_ (*i.v.*); Group 4, MPTP + SOD (*i.v.*); Group 5, MPTP + SOD@EXO (*i.v.*); Group 6, MPTP + SOD@EXO + CeO_2_ (*i.v.*); Group 7, MPTP + SOD@EXO + CeO_2_ (*i.n.*). Except for the mice of Group 1, others were intraperitoneally injected with MPTP for consecutive 5 days to induce the *in vivo* PD mice model (Fig. 4A). Motor function is widely known as a neurological indicator for the evaluation of PD. To explore the therapeutic effect in the PD mice, climbing pole test was performed every other day from Day 4 and the locomotor time climbing from one side of pole to the other was measured. Compared with the untreated healthy mice, the pole climbing time of MPTP group significantly increased owing to the PD progression (Fig. 4B). In comparison to the intravenous injection of SOD@EXO + CeO_2_, the intranasal administration group showed greater advantages with shorter climbing pole time on Day 16 (4.5 ± 1.32 s *vs* 3.83 ± 0.76 s), owing to the higher accumulation of SOD@EXO + CeO_2_ in brain after the intranasal administration. As shown in Fig. S10A, the time on rotarod was significantly shorter for the MPTP-induced PD mice than healthy ones, indicating the severe deficits in motor coordination and balance. The treatment with SOD@EXO + CeO_2_ could significantly prolong the retention time on the rotarod. Similarly, the asymmetry index of SOD@EXO + CeO_2_ (*i.v.*) and SOD@EXO + CeO_2_ (*i.n.*) groups was much lower than the MPTP-induced PD mice group (Fig. S10B). In addition, the total travel distance was reduced in the MPTP-induced PD model group compared with the untreated healthy mice (Fig. S10C). Notably, SOD@EXO + CeO_2_ increased the total travel distance, with a more pronounced improvement in the SOD@EXO + CeO_2_ (*i.n.*) group. Collectively, these behavioral tests demonstrated that SOD@EXO + CeO_2_ (*i.n.*) could protect the dopaminergic neurons and ameliorate the behavior disorder.

Further, the DA level, SOD activity, ROS level and MDA content were investigated to evaluate the therapeutic efficacy of different formulations. The DA level in the striatum of healthy mice was much higher than those treated with MPTP, and free SOD or CeO_2_ nanozyme did not significantly alter the DA level compared to the control group (Fig. 4C). Nevertheless, the DA level in SOD@EXO group was higher than that of native SOD group, indicating SOD@EXO could efficiently cross BBB for the PD treatment. As expected, the mice intravenously or intranasally treated with SOD@EXO + CeO_2_ showed obviously increasing DA levels. Similarly, high SOD activity was achieved after the SOD@EXO + CeO_2_ treatment owing to the successful transportation of SOD molecules into the brain (Fig. 4D). MDA was then measured in SNpc, which is a stable product of lipid peroxidation and a biomarker of oxidative stress [47]. As shown in Fig. 4E and F, the ROS level and MDA content of mice after the MPTP treatment were higher than those of untreated healthy group, indicating that MPTP could induce the long-term oxidative stress. For SOD@EXO + CeO_2_ (*i.n.*) group, the ROS level and MDA content in SNpc were much lower than the mice after the intravenous administration**.** The superior neuroprotection of SOD@EXO + CeO_2_ (*i.n.*) against the oxidative stress was mainly caused by the higher brain distribution of exosomes after the intranasal administration, thereby achieving favorable enzymatic activity in the brain.

### 3.6 In vivo anti-inflammatory efficacy of SOD@EXO + CeO_2_

Inflammatory responses of many neurodegenerative diseases are usually upregulated in the brain. MPTP could induce specific pathological changes in the loss of dopaminergic neurons in SNpc, thereby triggering the inflammatory responses and exacerbating the PD progression. Ionized calcium binding adapter molecule 1 (Iba-1) is the marker of microglia, while glial fibrillary acidic protein (GFAP) is the marker of astrocytes [48]. Except for these two factors, tumor necrosis factor-α (TNF-α), interleukin 1β (IL-1β) and interleukin 6 (IL-6) were also examined using qPCR analysis. As shown in Fig. 4G–K, the MPTP-induced PD mice significantly improved the secretion of inflammatory cytokines in comparison to Group 1 (the healthy C57BL/6 mice), whereas SOD@EXO inhibited the production of inflammatory cytokines in comparison to free SOD. Notably, the mRNA levels of inflammatory cytokines (TNF-α, IL-1β, IL-6, GFAP and Iba-1) were downregulated after the intravenous or intranasal administration of SOD@EXO + CeO_2_ in comparison to Group 2 (the MPTP-treated mice), suggesting the favorable neuronal protection effect mediated by the combination of SOD and CeO_2_ nanozyme. To check the treatment response at the tissue level, the mid-brains were processed into paraffin-embedded slides for the immunohistochemical analysis (Fig. 4L). As a biomarker of dopaminergic neurons, tyrosine hydroxylase completely degenerated in the MPTP-induced group compared to the healthy mice, which was in accordance with the pathological characteristics of PD. The expression of tyrosine hydroxylase recovered remarkably after the intravenous or intranasal administration of SOD@EXO + CeO_2_, which was consistent with the results using the MPP^+^-induced PD cell model. Besides, the semi-quantitative analysis of TH-positive neurons in the SNpc region was conducted using SaiViewer software (Fig. S11). The number of TH-positive neurons in the MPTP-induced PD model group was much lower than that of healthy mice. SOD@EXO + CeO_2_ treatment remarkably prevented the loss of TH-positive neurons, with no significant differences between the intravenous and intranasal administration. Collectively, these data supported that SOD@EXO + CeO_2_ inhibited the expression of neuroinflammatory cytokines and rescued the dopaminergic neurons. Finally, hematoxylin and eosin (H&E) staining of major organs (heart, liver, spleen, lungs and kidneys) was conducted to systematically investigate the safety of SOD@EXO + CeO_2_. Histopathology images revealed that no apparent damage to major tissues could be observed after the administration of various formulations (Fig. S12). In addition, Ernst et al. explored the long-term tissue accumulation and excretion of CeO_2_ nanoparticles, which indicated that the concentration of cerium decayed exponentially, accounting for about 50% excretion of cerium from the body in 100 days [49]. Importantly, no weight loss or apathy was observed, and CeO_2_ nanoparticles appeared safe without damaging the target organs. Nissl staining of brain tissues revealed a significant loss of Nissl bodies in the MPTP-treated PD mice compared to the healthy group (Fig. S13). In contrast, the SOD@EXO + CeO_2_ (*i.n.*) group showed an obvious preservation of neuronal morphology, with abundant Nissl bodies. These findings suggested that the treatment of SOD@EXO + CeO_2_ *via* intranasal administration could be potentially used as a promising and safe choice for chronic disorders that necessitated long-term therapeutic intervention.

## 4 Conclusions

In summary, the intranasal administration of CAT-like CeO_2_ nanozyme and SOD@EXO has been successfully established for the PD therapy. Native SOD was firstly incorporated into exosomes to obtain SOD@EXO, and then CeO_2_ nanozyme was mixed to obtain the formulation SOD@EXO + CeO_2_. The system SOD@EXO + CeO_2_ not only maintained ideal SOD activity but also exhibited high CAT activity. Meanwhile, the formulation could effectively scavenge the intracellular ROS level and alleviate the MPP^+^-induced cell death. Moreover, the non-invasive intranasal administration of exosomes increased the accumulation of cargos in brain compared to the intravenous injection, showing the advantage of intranasal administration in brain-targeting ability. Finally, the intranasal injection of SOD@EXO + CeO_2_ could alleviate the behavioral characteristics and pathological change of nerve injury in the MPTP-induced PD mice model. Additionally, SOD@EXO + CeO_2_ with desirable brain accumulation did not induce obvious systemic toxicity, demonstrating that the intranasal administration was safe and effective. As herein described, our exosome-related intranasal administration of native enzyme and nanozyme provided a new paradigm for constructing the prevention and treatment strategy of CNS dyskinesia disease.

## CRediT authorship contribution statement

**Xinxin Shao:** Writing – original draft, Software, Methodology, Investigation, Formal analysis, Data curation, Conceptualization. **Kefei Li:** Visualization, Validation, Resources, Investigation, Data curation. **Yanbo Wang:** Validation, Methodology, Investigation, Formal analysis, Data curation. **Xinai He:** Software, Methodology, Investigation, Formal analysis, Data curation. **Boyu Xiong:** Methodology, Formal analysis, Data curation. **Shengcai Yang:** Writing – review & editing, Supervision, Project administration, Funding acquisition. **Quanshun Li:** Writing – review & editing, Supervision, Funding acquisition, Conceptualization.

## Declaration of competing interests

The authors declare that they have no known competing financial interests or personal relationships that could have appeared to influence the work reported in this paper.

## Data Availability

Data will be made available on request.

## References

[1] Ben-Shlomo Y., Darweesh S., Llibre-Guerra J., Marras C., San Luciano M., Tanner C. (2024). The epidemiology of Parkinson's disease. Lancet.

[2] Morris H.R., Spillantini M.G., Sue C.M., Williams-Gray C.H. (2024). The pathogenesis of Parkinson's disease. Lancet.

[3] Burbulla L.F., Song P., Mazzulli J.R., Zampese E., Wong Y.C., Jeon S., Santos D.P., Blanz J., Obermaier C.D., Strojny C., Savas J.N., Kiskinis E., Zhuang X., Krüger R., Surmeier D.J., Krainc D. (2017). Dopamine oxidation mediates mitochondrial and lysosomal dysfunction in Parkinson's disease. Science.

[4] Fan C., Wei K., Chiu N., Liao E., Wang H., Wu R., Ho Y., Chan H., Wang T.A., Huang Y., Hsieh T., Lin C., Lin Y., Yeh C. (2021). Sonogenetic-based neuromodulation for the amelioration of Parkinson's disease. Nano Lett..

[5] Barnham K.J., Masters C.L., Bush A.I. (2004). Neurodegenerative diseases and oxidative stress. Nat. Rev. Drug Discov..

[6] Cheng G., Liu Y., Ma R., Cheng G., Guan Y., Chen X., Wu Z., Chen T. (2022). Anti-Parkinsonian therapy: strategies for crossing the blood-brain barrier and nano-biological effects of nanomaterials. Nano-Micro Lett..

[7] Xu J., Dai P., Zhang C., Dong N., Li C., Tang C., Jin Z., Lin S., Ye L., Sun T., Jin Y., Wu F., Luo L., Wu P., Li S., Li X., Hsu S., Jiang D., Wang Z. (2025). Injectable hierarchical bioactive hydrogels with fibroblast growth factor 21/edaravone/caffeic acid asynchronous delivery for treating Parkinson's disease. Adv. Sci..

[8] Wang W., Zheng J., Zhou H., Liu Q., Jia L., Zhang X., Ge D., Shi W., Sun Y. (2022). Polydopamine-based nanocomposite as a biomimetic antioxidant with a variety of enzymatic activities for Parkinson's disease. ACS Appl. Mater. Interfaces.

[9] Jiang L., Zhang X., Wang S., Zhang J., Chen J., Lu J., Yao L., Jin W., Li N., Li Q. (2025). Functional monomers equipped microgel system for managing Parkinson's disease by intervening chemokine axis-mediated nerve cell communications. Adv. Sci..

[10] Liu Y., Mao Y., Xu E., Jia H., Zhang S., Dawson V.L., Dawson T.M., Li Y., Zheng Z., He W., Mao X. (2021). Nanozyme scavenging ROS for prevention of pathologic α-synuclein transmission in Parkinson's disease. Nano Today.

[11] Liu J., Zhao T., Tan H., Cheng Y., Cao J., Wang F. (2010). Pharmacokinetic analysis of in vivo disposition of heparin-superoxide dismutase. Biomed. Pharmacother..

[12] Suzuki Y., Matsumoto T., Okamoto S., Hibi T. (2008). A lecithinized superoxide dismutase (PC-SOD) improves ulcerative colitis. Colorectal Dis..

[13] Cheng F., Kotha S., Fu M., Yang Q., Wang H., He W., Mao X. (2024). Nanozyme enabled protective therapy for neurological diseases. Nano Today.

[14] Jiang C., Shi Q., Yang J., Ren H., Zhang L., Chen S., Si J., Liu Y., Sha D., Xu B., Ni J. (2024). Ceria nanozyme coordination with curcumin for treatment of sepsis-induced cardiac injury by inhibiting ferroptosis and inflammation. J. Adv. Res..

[15] Esch F., Fabris S., Zhou L., Montini T., Africh C., Fornasiero P., Comelli G., Rosei R. (2005). Electron localization determines defect formation on ceria substrates. Science.

[16] Fu S., Chen H., Yang W., Xia X., Zhao S., Xu X., Ai P., Cai Q., Li X., Wang Y., Zhu J., Zhang B., Zheng J.C. (2022). ROS-targeted depression therapy via BSA-incubated ceria nanoclusters. Nano Lett..

[17] Yuan B., Tan Z., Guo Q., Shen X., Zhao C., Chen J.L., Peng Y. (2023). Regulating the H_2_O_2_ activation pathway on a well-defined CeO_2_ nanozyme allows the entire steering of its specificity between associated enzymatic reactions. ACS Nano.

[18] Gu Z., Zhong D., Hou X., Wei X., Liu C., Zhang Y., Duan Z., Gu Z., Gong Q., Luo K. (2024). Unraveling ROS conversion through enhanced enzyme-like activity with copper-doped cerium oxide for tumor nanocatalytic therapy. Adv. Sci..

[19] Lian X., Erazo-Oliveras A., Pellois J., Zhou H. (2017). High efficiency and long-term intracellular activity of an enzymatic nanofactory based on metal-organic frameworks. Nat. Commun..

[20] Zhang X., Gu Y., Liu Y., Cai Y., Sun H., Liu Y., Liu M. (2024). Near-infrared enhanced organic Se-doped carbon nitride quantum dots nanozymes as SOD/CAT mimics for anti-Parkinson via ROS-NF-κB-NLRP3 inflammasome axis. Chem. Eng. J..

[21] Chapman R., Stenzel M.H. (2019). All wrapped up: stabilization of enzymes within single enzyme nanoparticles. J. Am. Chem. Soc..

[22] Qin X., Yu C., Wei J., Li L., Zhang C., Wu Q., Liu J., Yao S.Q., Huang W. (2019). Rational design of nanocarriers for intracellular protein delivery. Adv. Mater..

[23] Colombo M., Raposo G., Thery C. (2014). Biogenesis, secretion, and intercellular interactions of exosomes and other extracellular vesicles. Annu. Rev. Cell Dev. Biol..

[24] Cocozza F., Grisard E., Martin-Jaular L., Mathieu M., Théry C. (2020). SnapShot: extracellular vesicles. Cell.

[25] Shao X., Feng X., Feng J., Qiu T., Yang S., Li Q. (2025). Exosome-mediated co-delivery of superoxide dismutase and chondroitinase ABC for multiple sclerosis therapy. Int. J. Biol. Macromol..

[26] Li Z., Zhou X., Wei M., Gao X., Zhao L., Shi R., Sun W., Duan Y., Yang G., Yuan L. (2019). In vitro and in vivo RNA inhibition by CD9-HuR functionalized exosomes encapsulated with miRNA or CRISPR/dCas9. Nano Lett..

[27] Luan X., Sansanaphongpricha K., Myers I., Chen H., Yuan H., Sun D. (2017). Engineering exosomes as refined biological nanoplatforms for drug delivery. Acta Pharmacol. Sin..

[28] Rehman F.U., Liu Y., Zheng M., Shi B. (2023). Exosomes based strategies for brain drug delivery. Biomaterials.

[29] Perets N., Hertz S., London M., Offen D. (2018). Intranasal administration of exosomes derived from mesenchymal stem cells ameliorates autistic-like behaviors of BTBR mice. Mol. Autism.

[30] Peng H., Li Y., Ji W., Zhao R., Lu Z., Shen J., Wu Y., Wang J., Hao Q., Wang J., Wang W., Yang J., Zhang X. (2022). Intranasal administration of self-oriented nanocarriers based on therapeutic exosomes for synergistic treatment of Parkinson's disease. ACS Nano.

[31] González L.F., Acuña E., Arellano G., Morales P., Sotomayor P., Oyarzun-Ampuero F., Naves R. (2021). Intranasal delivery of interferon-β-loaded nanoparticles induces control of neuroinflammation in a preclinical model of multiple sclerosis: a promising simple, effective, non-invasive, and low-cost therapy. J. Control. Release.

[32] Haney M.J., Klyachko N.L., Zhao Y., Gupta R., Plotnikova E.G., He Z., Patel T., Piroyan A., Sokolsky M., Kabanov A.V., Batrakova E.V. (2015). Exosomes as drug delivery vehicles for Parkinson's disease therapy. J. Control. Release.

[33] Han S.I., Lee S., Cho M.G., Yoo J.M., Oh M.H., Jeong B., Kim D., Park O.K., Kim J., Namkoong E., Jo J., Lee N., Lim C., Soh M., Sung Y., Yoo J., Park K., Hyeon T. (2020). Epitaxially strained CeO_2_/Mn_3_O_4_ nanocrystals as an enhanced antioxidant for radioprotection. Adv. Mater..

[34] Kwon H.J., Cha M., Kim D., Kim D.K., Soh M., Shin K., Hyeon T., Mook-Jung I. (2016). Mitochondria-targeting ceria nanoparticles as antioxidants for Alzheimer's disease. ACS Nano.

[35] Shao X., Zhang M., Chen Y., Sun S., Yang S., Li Q. (2023). Exosome-mediated delivery of superoxide dismutase for anti-aging studies in *Caenorhabditis elegans*. Int. J. Pharm..

[36] Cunha S., Forbes B., Lobo J.M.S., Silva A.C. (2021). Improving drug delivery for Alzheimer's disease through nose-to-brain delivery using nanoemulsions, nanostructured lipid carriers (NLC) and in situ hydrogels. Int. J. Nanomed..

[37] Wang S., He H., Chen L., Zhang W., Zhang X., Chen J. (2015). Protective effects of salidroside in the MPTP/MPP^+^-induced model of Parkinson's disease through ROS-NO-related mitochondrion pathway. Mol. Neurobiol..

[38] Cai Y., Ren W., Du C., Zhang J., Zhao Y., Yu J., Du H., Kang H., Ge X., Lu M., Li S., Jiang T. (2025). Cerium oxide nanozymes: exploring synthesis, catalytic activity, and biomedical potential. Small.

[39] Mohajeri M., Momenai R., Mohajeri S.K., Ohadi M., Estabragh M.A.R. (2025). Cerium oxide nanoparticles, physical and chemical properties, applications and toxicological implications: a review. Results Chem..

[40] Cassee F.R., van Balen E.C., Singh C., Green D., Muijser H., Weinstein J., Dreher K. (2011). Exposure, health and ecological effects review of engineered nanoscale cerium and cerium oxide associated with its use as a fuel additive. Crit. Rev. Toxicol..

[41] Berret J.F., Sehgal A., Morvan M., Sandre O., Vacher A., Airiau M. (2006). Stable oxide nanoparticle clusters obtained by complexation. J. Colloid Interface Sci..

[42] Yang J., Xiao S., Deng J., Li Y., Hu H., Wang J., Lu C., Li G., Zheng L., Wei Q., Zhong J. (2024). Oxygen vacancy-engineered cerium oxide mediated by copper-platinum exhibit enhanced SOD/CAT-mimicking activities to regulate the microenvironment for osteoarthritis therapy. J. Nanobiotechnol..

[43] Chen C.C., Liu L., Ma F., Wong C.W., Guo X.E., Chacko J.V., Farhoodi H.P., Zhang S.X., Zimak J., Ségaliny A., Riazifar M., Pham V., Digman M.A., Pone E.J., Zhao W. (2016). Elucidation of exosome migration across the blood-brain barrier model in vitro. Cell. Mol. Bioeng..

[44] Lin M.T., Beal M.F. (2006). Mitochondrial dysfunction and oxidative stress in neurodegenerative diseases. Nature.

[45] Itokazu Y., Fuchigami T., Morgan J.C., Yu R.K. (2021). Intranasal infusion of GD3 and GM1 gangliosides downregulates alpha-synuclein and controls tyrosine hydroxylase gene in a PD model mouse. Mol. Ther..

[46] Xie X., Song Q., Dai C., Cui S., Tang R., Li S., Chang J., Li P., Wang J., Li J., Gao C., Chen H., Chen S., Ren R., Gao X., Wang G. (2023). Clinical safety and efficacy of allogenic human adipose mesenchymal stromal cells-derived exosomes in patients with mild to moderate Alzheimer's disease: a phase I/II clinical trial. Gen. Psych..

[47] Bai S., Shao X., Tao Y., Wang S., Han H., Li Q. (2022). Superoxide dismutase-embedded metal–organic frameworks via biomimetic mineralization for the treatment of inflammatory bowel disease. J. Mater. Chem. B.

[48] Lee J., Ahn J.H., Lee D.H., Yan B.C., Park J.H., Kim I.H., Cho G., Kim Y., Lee B., Park C.W., Cho J.H., Lee H.Y., Won M. (2013). Neuronal damage and gliosis in the somatosensory cortex induced by various durations of transient cerebral ischemia in gerbils. Brain Res..

[49] Ernst L.M., Mondragón L., Ramis J., Gustà M.F., Yudina T., Casals E., Bastús N.G., Fernández-Varo G., Casals G., Jiménez W., Puntes V. (2023). Exploring the long-term tissue accumulation and excretion of 3 nm cerium oxide nanoparticles after single dose administration. Antioxidants.

