# Supplementary material for: Intranasal delivery of cerium oxide nanozyme and superoxide dismutase in exosomes for Parkinson's disease therapy
Source: Mater Today Bio. 2026 Sep 2;40:103629. doi: 10.1016/j.mtbio.2026.103629 (PMC13579301; doi:10.1016/j.mtbio.2026.103629)
Supplement: Supplementary file 1 [file mmc1.docx]

**Supporting Information**

Intranasal Delivery of Cerium Oxide Nanozyme and Superoxide Dismutase in Exosomes for Parkinson’s Disease Therapy

Xinxin Shao,^1^ Kefei Li,^1^ Yanbo Wang, Xinai He, Boyu Xiong, Shengcai Yang*, Quanshun Li*

*Key Laboratory for Molecular Enzymology and Engineering of Ministry of Education, School of Life Sciences, Jilin University, Changchun 130012, China*

*Corresponding author.

Tel. and Fax: +86-431-85155200.

E-mail: [yang_shengcai@jlu.edu.cn](mailto:yang_shengcai@jlu.edu.cn) (S. Yang); [quanshun@jlu.edu.cn](mailto:quanshun@jlu.edu.cn) (Q. Li).

^1^These authors contributed equally to the work.

**Materials and methods**

*1.1. Cell culture*

Human embryonic kidney cell line HEK293, human neuroblastoma cell line SH-SY5Y and human umbilical vein endothelial cell line HUVEC were purchased from Shanghai Institute of Cell Bank (Shanghai, China). HEK293 and SH-SY5Y cells were cultured in DMEM supplemented with 10% FBS and 1% penicillin/streptomycin at 37 °C in a 5% CO_2_ atmosphere. HUVEC cells were cultured in RPMI 1640 medium supplemented with 10% FBS and 1% penicillin/streptomycin at 37 °C in a 5% CO_2_ atmosphere.

*1.2. Isolation and characterization of exosomes*

The HEK293-derived exosomes were isolated by differential centrifugation. In brief, FBS was ultracentrifuged at 100,000×*g* for 1.5 h to remove original exosomes and obtain exosome-depleted FBS. HEK293 cells were cultured to 60-70% confluence in 10% FBS-containing DMEM, washed with PBS and incubated in fresh DMEM harboring 10% exosome-depleted FBS for 48 h. Afterwards the collected medium was centrifuged at 300×*g* for 5 min to discard dead cells and subjected to the centrifugation at 2,000×*g* for 10 min and 10,000×*g* for 30 min to eliminate the cell debris. The supernatant was then collected and filtered using a 0.22-μm filter (Millipore, Merck, Germany) to remove residual cells and cell debris. Finally, the filtered supernatant was ultra-centrifuged at 100,000×*g* for 90 min (Beckman Coulter, Brea, CA, USA), and the precipitate was resuspended in PBS to obtain exosomes. All the centrifugation steps were performed at 4 °C.

*1.3. X-ray diffraction (XRD) analysis*

XRD analysis of liquid samples (CeO_2_, SOD@EXO and SOD@EXO+CeO_2_) was performed using an XRDynamic 500 X-ray diffractometer (Anton Paar, Austria), which was equipped with a Cu-Kα radiation source (λ = 0.15418 nm). The generator was operated at a tube voltage of 40 kV and a tube current of 40 mA. The liquid sample was injected into a dedicated liquid sample cell to ensure a flat surface without air bubbles. Diffraction patterns were recorded over a 2θ range of 10°-80° at a scanning speed of 2°/min with a step size of 0.02°. Background subtraction and peak position calibration were carried out using Materials Data Jade 6 software.

*1.4. Cellular uptake analysis*

The SH-SY5Y cells were seeded onto the sterilized coverslips placed in 6-well plates (1.8×10^5^ cells/well) and incubated to 70% confluence. SOD and exosomes were labeled with FITC and DID according to manufacturers’ instructions to obtain FITC-SOD and DID-EXO, respectively. FITC-SOD-loaded DID-EXO (FITC-SOD@DID-EXO) were added into the plates and incubated at 37 °C for 1, 2, 4 or 6 h. The medium was removed at predetermined time, and SH-SY5Y cells were stained with DAPI for 10 min to visualize the nucleus. The cellular uptake of FITC-SOD@DID-EXO was visualized by a confocal laser scanning microscope (CLSM, Carl Zeiss MicroImaging GmbH, Jena, Germany), and the images were analyzed by ZEN lite software.

*1.5. Cellular ROS level analysis*

The SH-SY5Y cells were inoculated in a 6-well plate (density: 8×10^5^ cells/well) and incubated overnight. CeO_2_ nanozyme (25 μg/mL), native SOD (6 U/mL), SOD@EXO (6 U/mL on SOD basis) and SOD@EXO+CeO_2_ (6 U/mL on SOD basis) were used to treat the cells for 6 h, which were then treated with MPP^+^ (2 mM) for 2 h. After the treatment with DCFH-DA probe (10 μM) in the ROS assay kit (S0033, Beyotime, China) at 37 °C for 20 min in the dark, the fluorescent intensity was measured by CytoFLEX flow cytometry (Beckman Coulter, IN, USA) and analyzed by CytExpert software.

*1.6. Cell viability assay*

The cell viability was evaluated based on the MTT assay. The SH-SY5Y cells were seeded in a sterile 96-well plate at a density of 8×10^3^ cells/well and incubated overnight for the cell attachment. To evaluate the biocompatibility of CeO_2_ nanozyme, the cells were incubated with different concentrations of CeO_2_ nanozyme (0, 10, 25, 50, 100 and 250 μg/mL) at 37 °C for 24 h. Subsequently, 20 μL of MTT solution (5 mg/mL) was added into the 96-well plate and incubated for another 4 h. The medium was removed, and 200 μL DMSO was used to dissolve the formazan for 15 min. The optical density at 570 nm was measured by GF-M3000 microplate reader (Shandong, China). The cell viability (%) was calculated according to the following equation:

Cell viability (%) = (A_treated_-A_blank_)/(A_untreated_-A_blank_) × 100%

in which A_treated_ and A_untreated_ represented the optical density of treated cells and untreated cells, respectively, and A_blank_ was the absorbance of medium to calibrate the reads.

In addition, the neuroprotective ability of CeO_2_ nanozyme, SOD, SOD@EXO and SOD@EXO+CeO_2_ was evaluated using a similar method, in which CeO_2_ nanozyme (25 μg/mL), SOD (6 U/mL), SOD@EXO (6 U/mL on SOD basis) and SOD@EXO+CeO_2_ (6 U/mL on SOD basis) were used to pretreat the cells for 6 h. To construct the PD cell model, the cells were treated with 2 mM MPP^+^ solution for 24 h.

*1.7. Western blotting analysis*

The SH-SY5Y cells were seeded in a 6-well plate at a density of 1.8×10^5^ cells/well and incubated overnight for the cell attachment. Then, CeO_2_ nanozyme (25 μg/mL), native SOD (6 U/mL), SOD@EXO (6 U/mL on SOD basis) and SOD@EXO+CeO_2_ (6 U/mL on SOD basis) were incubated with the cells for 6 h, which were subsequently treated with MPP^+^ (2 mM) for 24 h. The cells were lysed with radioimmunoprecipitation assay (RIPA) lysis buffer containing 1% phenylmethylsulfonyl fluoride for 1 h on ice and centrifuged at 2,000×*g* at 4 °C for 15 min. The supernatants were collected, and the protein concentration was measured using bicinchoninic acid (BCA) protein assay kit. 50 μg of extracted proteins was analyzed using 12% (w/v) SDS-PAGE and transferred to polyvinylidene difluoride membrane (Billerica, MA, USA) via electroblotting. The membrane was blocked in non-fat milk solution (5%) at room temperature for 3 h and incubated with primary antibodies at 4 °C for 12 h, in which β-actin was served as an internal reference. The primary antibodies included anti-procaspase-3 antibody (1:10000, Abcam, ab32499), anti-procaspase-9 antibody (1:1000, Abcam, ab138412), anti-Bcl-2 antibody (1:1000, Abcam, ab196495), anti-tyrosine hydroxylase antibody (1:500, Abcam, ab6211), anti-α-synuclein antibody (1:5000, Abcam, ab51253) and anti-β-actin antibody (1:5000, Abcam, ab8226). Blots were then incubated with horseradish peroxidase (HRP)-conjugated secondary antibodies at room temperature for 2 h. Protein bands were detected by EasySee enhanced chemical luminescence (TransGen, Beijing, China) and analyzed using the ImageJ software (National Institute of Health, USA). Similarly, exosomes were resuspended in RIPA lysis buffer and the biomarkers were determined by Western blotting using the primary antibodies as follows: anti-SOD antibody (1:5000, Abcam, ab13498), anti-TSG101 antibody (1:1000, Abcam, ab125011) and anti-CD63 antibody (1:400, Abcam, ab193349).

*1.8. Cellular SOD activity analysis*

The SH-SY5Y cells were treated as described in the section “Western blotting analysis”. After the lysis of cells using RIPA buffer, the supernatant was collected by the centrifugation at 3,000×*g* for 15 min. The cellular SOD activity was analyzed with superoxide dismutase activity assay kit (SOD, S0101, Beyotime, China) according to the manufacturer’s instructions.

*1.9. Live/dead cell staining*

Live/dead cell staining assay was performed using LIVE/DEAD^®^ Viability/Cytotoxicity kit (ethidium homodimer 1 (EthD-1) and calcein AM). Briefly, SH-SY5Y cells were treated as described in the section “Western blotting analysis”. After the treatment with different groups and MPP^+^, the cells were stained with calcein AM (2.0 mM) and EthD-1 (4.0 mM) at 37 °C for 30 min and observed on Olympus IX71 fluorescence microscope (Tokyo, Japan).

*1.10. Cell apoptosis analysis*

For the cell apoptosis assay, SH-SY5Y cells were treated as described in the section “Western blotting analysis”. After the treatment with MPP^+^ (2 mM) for 24 h, the detached and trypsinized cells were collected and centrifuged at 2,000 rpm for 10 min, and treated with Annexin V-FITC and PI according to the manufacturer’s protocol. The cell apoptosis was detected by CytoFLEX flow cytometry (Beckman Coulter, IN, USA) and analyzed by CytExpert software.

*1.11. Mitochondrial membrane potential detection*

The mitochondrial membrane potential was measured using the fluorescent probe JC-1, in which JC-1 aggregated and emitted red fluorescence under high mitochondrial membrane potential, while it existed as monomers and exhibited green fluorescence at lower potential values. In short, SH-SY5Y cells were treated as described in the section “Western blotting analysis”. After the treatment with different samples, the cells were stained with JC-1 dye for 30 min according to the guideline in the mitochondrial membrane potential detection kit, and the images were captured by Olympus IX71 fluorescence microscope (Tokyo, Japan).

*1.12. Immunofluorescence analysis for the expression level of tyrosine hydroxylase*

The SH-SY5Y cells were treated as described in the section “Western blotting analysis” after the adherence to the plate, and then rinsed with PBS and fixed in 4% paraformaldehyde at room temperature for 20 min. Subsequently, the cells were permeabilized with Triton X-100 (0.1%) for 15 min, blocked in 3% goat serum for 30 min, and incubated with the primary antibody against tyrosine hydroxylase at 4 °C overnight. After washing with PBST solution (PBS containing 0.05% Tween 20) three times, the cells were incubated with the secondary antibody at room temperature for 1 h in the dark, stained with DAPI and visualized by Olympus IX71 fluorescence microscope (Tokyo, Japan).

*1.13. Rotarod test, cylinder test and open field test in MPTP-induced PD mice model*

Male C57BL/6 mice were randomly divided to 4 groups (n = 10 per group): untreated healthy mice (Normal); MPTP + Control; MPTP + SOD@EXO+CeO_2_ (*i.v.*); MPTP + SOD@EXO+CeO_2_ (*i.n.*). The administration time and dosage were consistent with those described in the section 2.6. The open field test was used to evaluate the locomotion behavior. On Day 14, the mice were placed at the center of a plastic square box (40 × 40 × 40 (cm)) and allowed to freely explore for 10 min under dim light conditions (approximately 15 lux) with white noise playing to minimize environmental distractions. The movement of the mice was recorded, and the total travel distance was analyzed using Smart V3.0 video tracking system (Panlab).

The rotarod test was performed to evaluate the motor coordination and balance ability on Day 15. The mice underwent the training on Day 13 and 14. The mice were placed on the YSL-4 Rotarod (Yiyan Science & Technology Development Co. Ltd., Jinan, China) and trained at a constant speed of 4 rpm for 5 min, followed by a further 5 min of training in accelerating mode, ranging from 4 rpm to 40 rpm. Once the mice fell off the rod, they were immediately placed back onto the rotarod. On Day 15, the rotational speed was accelerated uniformly from 4 rpm to 40 rpm over a period of 5 min, and the time on rotarod was recorded.

The cylinder test was performed to assess the asymmetry index of forelimb use in mice on Day 16. Each mouse was individually placed into a transparent glass cylinder (10 cm in diameter and 20 cm in height). The duration of the test was 5 min. The number of supporting wall contacts performed with the right (R), the left (L) and both (B) forelimb was counted by an experimenter blinded to animal grouping. A forelimb-use asymmetry index was calculated using the following equation:

$$Asymmetry Index=\frac{L-R}{(L+R+B)} \times100$$

where L, R and B represented the number of supporting wall contacts performed with the right (R), the left (L) and both (B) forelimb, respectively.

*1.14. Immunohistochemical analysis*

The mice were sacrificed on Day 16, and the brains were extracted and fixed in 4% paraformaldehyde. The paraffin-embedded brain tissues were cut into 2-µm coronal sections and processed for the immunohistochemical analysis (IHC). Briefly, the brain sections were deparaffinized and dehydrated in xylene and a series of gradient ethanol. The tissue sections were soaked in EDTA antigen retrieval buffer (pH 9.0, G1203, Servicebio, Wuhan, China) and retrieved in a microwave oven. After cooling down to room temperature, the sections were placed in 3% H_2_O_2_ solution at room temperature for 25 min in the dark to eliminate the endogenous peroxidase activity. The sections were rinsed in PBS (pH 7.4) and incubated with 3% BSA for 30 min to avoid the non-specific binding. Afterward the sections were incubated with anti-tyrosine hydroxylase primary antibody (GB11181, 1:500, Servicebio, Wuhan, China) at 4 °C overnight, and treated with the HRP-conjugated secondary antibody (GB23303, 1:500, Servicebio, Wuhan, China) at 25 ^o^C for 50 min. Finally, the immunoreactions were visualized by diaminobenzidine tetrahydrochloride (G1212, Servicebio, Wuhan, China), and the nuclei of cells were counterstained with hematoxylin. The images were captured using Nikon DS-U3 camera (Tokyo, Japan). The number of TH-positive neurons in the SNpc region was counted using SaiViewer software (Servicebio, Wuhan, China).

*1.15. Hematoxylin* *and eosin (H&E) staining*

At the end of the treatment (Day 16), major organs (heart, liver, spleen, lung and kidney) were harvested from the mice and fixed in 4% paraformaldehyde at 4 °C for 24 h. Tissues were dehydrated and embedded in paraffin, and then the samples were cut into 7 μm-thick sections. The tissue slices were deparaffinized, rehydrated and stained with hematoxylin and eosin solution. Finally, the slices were visualized using Olympus IX71 fluorescence microscope (Tokyo, Japan).

*1.16. Nissl staining*

On Day 16, brain tissues were harvested from the mice and fixed in 4% paraformaldehyde. The tissues were dehydrated and embedded in paraffin, and then the samples were cut into 2-μm coronal sections. The tissue slices were deparaffinized and rehydrated in xylene and a series of gradient ethanol. The sections were stained with Nissl staining solution (0.5% Toluidine Blue, G1036, Servicebio, Wuhan, China) for 3 min, and rinsed with water. The dried sections were immersed in xylene for 10 min to achieve the transparency and then mounted with neutral gum.

*1.17. RNA isolation and quantitative* *real-time PCR (qPCR)*

Total RNAs were extracted from the SNpc homogenates with TRIzol reagent (TIANGEN, Beijing, China) and the concentration was quantified by Nano-600 spectrophotometer (JiaPeng Technology Co., Shanghai, China). Afterward, 1 μg of total RNA was used as a template to be reversely transcribed into the first-strand cDNA by PrimeScript RT reagent kit (TaKaRa, Dalian, China). Thereafter, qPCR was performed using the obtained cDNA samples with SYBR Premix Ex Taq kit, and β-actin was used as an internal control to quantitate the mRNA level. All the reactions were run in quadruplicate on Applied Biosystems 7500 Real-Time PCR Instrument (Foster City, CA, USA), and the mRNA level was calculated through 2^-ΔΔCt^ method. The primer sequences for qPCR analysis were used as follows:

Iba-1-forward: 5′-GTCCTTGAAGCGAATGCTGG-3′,

Iba-1-reverse: 5′-CATTCTCAAGATGGCAGATC-3′;

GFAP-forward: 5′-TCCTGGAACAGCAAAACAAG-3′,

GFAP-reverse: 5′-CAGCCTCAGGTTGGTTTCAT-3′;

IL-1β-forward: 5′-GCAACTGTTCCTGAACTCAACT-3′,

IL-1β-reverse: 5′-ATCTTTTGGGGTCCGTCAACT-3′;

IL-6-forward: 5′-CAATGGCAATTCTGATTGTATG-3′,

IL-6-reverse: 5′-AGGACTCTGGCTTTGTCTTTC-3′;

TNF-α-forward: 5′-CCCTCACACTCAGATCATCTTCT-3′,

TNF-α-reverse: 5′-GCTACGACGTGGGCTACAG-3′;

β-actin-forward: 5′-TCTGGCACCACACCTTCTACAATG-3′,

β-actin-reverse: 5′-GGATAGCACAGCCTGGATAGCAA-3′.

**Fig. S1.** The cell viability of SH-SY5Y cells after the incubation with CeO_2_ nanozyme for 24 h. Data were presented as mean value ± SD (n=6).

**Fig. S2.** Hydrodynamic diameter and PDI (A), and zeta potential (B) of EXO, SOD@EXO and SOD@EXO+CeO_2_. Data were presented as mean value ± SD (n=3).

**Fig. S3.** XRD analysis of CeO_2_ nanozyme, SOD@EXO and SOD@EXO+CeO_2_ in PBS.

**Fig. S4.** Hydrodynamic diameter and PDI of SOD@EXO+CeO_2_ in (A) PBS and (B) 10% FBS. Data were presented as mean value ± SD (n=3).

**Fig. S5.** (A) Western blotting analysis to determine the SOD concentration in SOD@EXO. Native SOD was used as standards (200, 400, 600, 800 and 1000 ng), and 3000 ng SOD@EXO was employed in the studies. Intensities of bands were calculated by Image J software to measure the SOD loading in exosomes. (B) The standard curve of band intensity of SOD in differernt concentration.

**Fig. S6.** SOD (A) and CAT (B) activity of CeO_2_ nanozyme and native SOD. Data were presented as mean value ± SD of triplicate experiments (n = 3, **P* < 0.05, ***P* < 0.01).

**Fig. S7.** Live/dead staining of SH-SY5Y cells after the pretreatment with different groups for 6 h in the MPP^+^-induced PD cell model. Scale bar: 200 μm.

**Fig. S8.** Flow cytometric analysis for the ROS level in SH-SY5Y cells after the pretreatment with different groups for 6 h in the MPP^+^-induced PD cell model.

**Fig. S9.** Quantification of the fluorescent intensity of brains at 1 h, 3 h and 6 h post-injection of free DIR, DIR-EXO (*i.v.*) and DIR-EXO (*i.n.*) (n=3, ***P* < 0.01).

**Fig. S10.** (A) The time on rotarod, (B) asymmetry index and (C) total travel distance of mice after different treatments (n = 10, **P* < 0.05, ***P* < 0.01, ****P* < 0.001, *****P* < 0.0001).

**Fig. S11.** The number of TH-positive neurons after different treatments (n = 3, ***P* < 0.01, ****P* < 0.001).

**Fig. S12.** H&E staining of major organs in different groups. Scale bars: 50 μm.

**Fig. S13.** Nissl staining of brain tissues in different groups. Scale bar: 100 μm.

**Table S1.** Hydrodynamic diameter, PDI and zeta potential of EXO, SOD@EXO and SOD@EXO+ CeO_2_. Data were presented as mean value ± SD of triplicate experiments.

| **Formulation** | **Hydrodynamic diameter (nm)** | **PDI** | **Zeta potential**  **(mV)** |
| --- | --- | --- | --- |
| EXO | 100.6 ± 0.9 | 0.22 ± 0.01 | -17.9 ± 0.6 |
| SOD@EXO | 96.8 ± 2.5 | 0.30 ± 0.02 | -22.1 ± 3.8 |
| SOD@EXO+CeO_2_ | 103.2 ± 1.7 | 0.24 ± 0.01 | -19.6 ± 3.7 |
